# Some Observations of Bird Life

**Published:** 1916-12-02

**Authors:** 


### NATURAL HISTORY NOTES FROM MACEDONIA.

### Some Observations of Bird Life.

By AN OFFICER OF THE R.A.M.C. AND THE AUTHOR OF "NATURE NOTES."

Bird-life on the Macedonian hills and plains is very various. Unfortunately I am not able to name many species I see. The kestrel is bound to attract the attention of any traveller, for this beautiful little hawk is plentiful and exceedingly confiding. It is a common sight to see six or eight of them hovering in a group over some reach of stubble. Seen closely, as they are here, the beauty of their plumage and the grace with which they sweep and glide across the wind are very charming. They throw themselves up to the wind and hover with tail depressed, wings flapping rapidly, and eyes turned down to survey the ground for prey. I believe they chiefly feed on grasshoppers. They often drop down to seize something; it is seldom I see a mouse in their claws, and never a bird, but something smaller they gather in. This can only be grasshoppers. Sometimes I see one make several dashes at something just above the ground as if prey missed sought to escape by flight.

In the ravines that cut across the country and have in them water and a lush growth of rushes, scrub, and willow-herb, and also in the marshy ground down by the lakes and rivers, a brown hawk, large, rather ragged-looking, and floppy of flight, is common. It rises in front of my horse as it pushes through the tangle of marsh growth, and flops away slowly close to the tops of the reeds. I believe it to be the marsh harrier, a bird once common in the English fens. Kites, vultures, and a nearly white scavenger with a yellow beak, common in India, all are plentiful. Already the forerunners of the passage falcons are putting in an appearance. I write this on September 4.

The number and variety of the crow tribe is very astonishing. Magpies are everywhere, often in flocks of twenty or thirty; jackdaws in huge flocks like rooks in England. Rooks do not exist here so far as I know. There are great numbers of the hooded crow, many carrion crows, and a goodly number of ravens. A very common bird is the roller, often called the blue jay on account of a superficial resemblance to that bird. It is a solitary and insectivorous bird of dull appearance as it sits motionless on some low bush, but when it opens its wings in flight it reveals a surprising and beautiful display of bright blue feathers. From a point of vantage, such as a bush or telegraph wire, it dashes suddenly down to catch its insect prey.

The hoopoe is very plentiful, but much less confiding than in India, where it is always in evidence on the drives of bungalows, delighting to disport itself in the white dust of *kunkur* roads. It nests in holes. A young bird kept by one of my field ambulances became very tame.

Storks are very plentiful. They have huge nests on the houses or in tall trees. When I arrived in Macedonia the young had left the nests, but the parent pairs none the less have not deserted their homes, for often a pair may be seen standing solemnly and still, side by side, as if ruminating on the fate of the offspring that had forsaken it. The nest appears to be the roosting-place. It is amusing to watch storks feeding. They stalk slowly across the fields, moving each leg with great deliberation, prodding long beaks into tufts of herbage. Suddenly the sight of a frightened frog trying to escape galvanises one of them into intense ungainly activity. With a sudden rush and high stepping of long legs the frog is caught and, despite struggles, manœuvred into a suitable position and engulfed.

Great numbers of tortoises exist both in plain and hill. It is scarcely possible to ride a couple of miles without coming on one or more. They are of all sizes, from little fellows lately hatched, soft of shell and scarcely larger than half an egg, to wrinkle-shelled veterans perhaps some centuries old, if popular rumour of their longevity may be believed, and near a foot in span. Even they are not unaffected by the turmoil of war. Many an old fellow deliberately crossing the road is caught and crushed by lorries moving at furious speed compared to that of the slow-going bullock wains that for generations have been his only experience of wheeled transport.

Some part of the French troops recognise the tortoise as an article of food. Many have gone to the pot. In July and August the males were pursuing the females with amorous intent. It was common to see a female speeding across country at the rate of half a mile an hour, with a train of two or three males dashing at similar speed after her. The male that reached her would bite her hind leg to make her stay her flight and woo her with gasped-out squeaks. There is much to interest the Nature-lover in Macedonia, but none the less I, as all of us, shall be glad when we can push forward and see more exciting scenes than the kestrels hovering for their prey and the amorous antics of the tortoise.

